# Language, Speech, and Facial Expression Features for Artificial Intelligence–Based Detection of Cancer Survivors’ Depression: Scoping Meta-Review

**DOI:** 10.2196/30439

**Published:** 2021-12-06

**Authors:** Urška Smrke, Izidor Mlakar, Simon Lin, Bojan Musil, Nejc Plohl

**Affiliations:** 1 Faculty of Electrical Engineering and Computer Science University of Maribor Maribor Slovenia; 2 Science Department Symptoma Vienna Austria; 3 Department of Internal Medicine Paracelsus Medical University Salzburg Austria; 4 Department of Psychology Faculty of Arts University of Maribor Maribor Slovenia

**Keywords:** artificial intelligence, cancer, depression, facial expression, language, oncology, review, screening, speech, symptom

## Abstract

**Background:**

Cancer survivors often experience disorders from the depressive spectrum that remain largely unrecognized and overlooked. Even though screening for depression is recognized as essential, several barriers prevent its successful implementation. It is possible that better screening options can be developed. New possibilities have been opening up with advances in artificial intelligence and increasing knowledge on the connection of observable cues and psychological states.

**Objective:**

The aim of this scoping meta-review was to identify observable features of depression that can be intercepted using artificial intelligence in order to provide a stepping stone toward better recognition of depression among cancer survivors.

**Methods:**

We followed a methodological framework for scoping reviews. We searched SCOPUS and Web of Science for relevant papers on the topic, and data were extracted from the papers that met inclusion criteria. We used thematic analysis within 3 predefined categories of depression cues (ie, language, speech, and facial expression cues) to analyze the papers.

**Results:**

The search yielded 1023 papers, of which 9 met the inclusion criteria. Analysis of their findings resulted in several well-supported cues of depression in language, speech, and facial expression domains, which provides a comprehensive list of observable features that are potentially suited to be intercepted by artificial intelligence for early detection of depression.

**Conclusions:**

This review provides a synthesis of behavioral features of depression while translating this knowledge into the context of artificial intelligence–supported screening for depression in cancer survivors.

## Introduction

While cancer incidence is increasing worldwide [1], so are 5-year survival rates, from 49% in the 1970s to 69% in 2017 [2,3]. After having cancer, individuals can be faced with a wide array of challenges, such as fatigue, pain, impaired cognitive functions, and fear of cancer recurrence [1]. Among these challenges, depressive spectrum and mood-related disorders are among the most common psychological conditions [4]; it is estimated that in the first 2 years after diagnosis, 12% to 20% of cancer survivors meet diagnostic criteria for major depression disorder [1,5,6]. This is even more pronounced in cancer survivors with other comorbid chronic diseases [7] and breast cancer survivors [8]. At any point during survivorship, a dysphoric mood, anxiety, appetite changes, insomnia, or irritability can present and last weeks or even months [9]; however, these conditions frequently remain underrecognized and overlooked in clinical practice [10] because the signs and symptoms of depression in patients with cancer are heterogeneous [11]. Many survivors are also more likely to report somatic complaints rather than an overtly depressed mood. Moreover, only a minority of posttreatment survivors report experiencing clinically significant psychological distress during the treatment phase of their disease [12]. Although cancer distress screening has been recognized as an important tool, health care systems struggle to implement these tools in practice [13]. Lack of staff, difficulty in differentiating between mental health distress and symptoms of the disease, time constraints, accessibility restrictions, and availability of services represent the major barriers to regular screening [14].

Patient-gathered health data and patient-reported outcomes, in particular, have become a valuable tool in understanding the symptomatology of cancer patients and survivors [15]. Patient-reported outcomes give voice to the patient’s perspective and can inform decision-making by providing insight into the quality of life that is complementary to clinician-rated adverse events [16]. Digital interventions that collect patient-reported outcomes have been recognized as feasible and acceptable by clinicians and patients alike [17]. However, self-reporting often involves reporting bias, which may result in erroneous judgments such as the reconstruction of memories and excessive reliance on cognitive heuristics [18]. Retrospective self-reports of negative mood states experienced in the past (eg, the most recent 2 weeks) tend to be exaggerated in a negative direction [19]. Reporting and interpretation biases are even more pronounced for people suffering from symptoms of depression [20,21]. Moreover, inattentive responding and social desirability may distort the quality of results even further [22,23]. Currently, big data and artificial intelligence offer new opportunities for the screening and prediction of mental health problems. Specifically, there is a growing interest in examining relationships between observable cues, such as language use, speech, and facial expressions, and the psychological characteristics of the communicator [24]. These observable cues are generated spontaneously, are less impacted by cognitive and other biases related to desirability and crassness, and can contribute to improving the objectivity of psychiatric assessments [25]. The main motivation of this scoping review was to identify the observable features of depressive symptoms that are expressed during conversation (often without awareness), and that can be intercepted using artificial intelligence to be developed in the project *Patient-Centered Survivorship Care Plan After Cancer Treatments Based on Big Data and Artificial Intelligence Technologies* (PERSIST [26]). To our knowledge, past reviews (eg, [25,27-29]) mainly focused on specific modalities (ie, text, language, vocal features, or facial expressions). None, however, has focused on integration and the analysis of the complementary role of these modalities when expressed concurrently during a conversation; therefore, in this scoping meta-review, we integrated information from systematic reviews and meta-analyses on specific modalities and observable features of depression that are capable of being exploited by artificial intelligence.

## Methods

### Overview

We followed the methodological framework outlined by Arksey and O’Malley [30] and Levac and colleagues [31]. The framework proposes 6 stages: (1) identifying the research questions; (2) identifying relevant studies; (3) study selection; (4) charting the data; (5) collating, summarizing, and reporting results; and (6) consultation exercises. To ensure the process of this scoping review was systematic, transparent, and complete, we followed PRISMA-ScR (Preferred Reporting Items for Systematic Reviews and Meta-Analyses extension for Scoping Reviews [32]) guidelines.

### Identifying the Research Questions

We first established the purpose of the review and developed specific research questions to guide our scoping review (eg, search terms, eligibility criteria)—which (1) text-related cues, (2) speech-related cues, and (3) facial expressions offer a valid insight into individuals’ depression?

### Identifying Relevant Studies

Two large and commonly used databases, SCOPUS and Web of Science, which complement each other well [33,34], were used to identify the relevant papers. After a preliminary search in both databases, which helped us to refine the search strategy and ensure that the databases provide adequate coverage of the research topic, we conducted the main search on March 16, 2021.

Our search strategy combined terms related to depression (depression, “major depressive disorder,” "depressive episode,” depressivity, depressed), cues (feature*, indicator*, marker*, sign, signs, signal, signals, cue*, symbol*, pattern*, style*, clue*, manifestation*, expression*), behavior (language, linguistic, speech, acoustic, “facial expression”), and reviews (review, meta-analysis, “state of the art,” state-of-the-art). It should be noted that the last group of keywords (ie, those pertaining to reviews) was added in the process of search strategy refinement, as the number of papers was otherwise too high and not feasible for a scoping review. As such, the present scoping review is a scoping meta-review, which uses high-level evidence to answer research questions and policy dilemmas [35]. Additionally, the search strategy included keywords related to different disorders that affect behavior and display of emotions (dementia, ”Parkinson's disease,” autism, schizophrenia, Alzheimer's, “neurological disorder,” stroke, “Huntington's disease,” paralysis, mutism, “multiple sclerosis,” “cerebral palsy,” “Down syndrome”). Since comorbidity of depression and these disorders could skew our conclusions, such papers were already excluded in the first step. These groups of keywords were later combined into a nested format using Boolean operators (AND, OR, NOT), and titles, abstracts, and keywords were searched. The exact search string for SCOPUS was “TITLE-ABS-KEY ( ( depression OR “major depressive disorder” OR “depressive episode” OR depressivity OR depressed ) AND ( sign OR signs OR signal OR signals OR cue* OR symbol* OR pattern* OR style* OR clue* OR manifestation* OR expression* OR feature* OR indicator* OR marker* ) AND ( speech OR “facial expression” OR language OR linguistic OR acoustic ) AND ( review OR meta-analysis OR “state of the art” OR state-of-the-art ) AND NOT ( dementia OR “Parkinson's disease” OR autism OR schizophrenia OR “Alzheimer's disease” OR “neurological disorder*” OR stroke OR “Huntington's disease” OR paralysis OR mutism OR “multiple sclerosis” OR “cerebral palsy” OR “Down syndrome” ) ).” In order to identify potential additional papers, different combinations of search terms were used in Google Scholar, because this database can lead to the identification of additional unique entries [36].

The inclusion criteria were derived from the research questions guiding this scoping review and were set a priori. In order to be considered for inclusion in the scoping review, studies had to (1) be available in English and (2) published (ie, preprints and other unpublished papers were not considered). Studies were then excluded if (1) they were not based on empirical primary studies (ie, no quantitative evidence, obtained by collecting data on human participants), (2) they included human participants who suffered from other disorders that affect behavior and display of emotions, (3) they did not focus on adults, and (4) they did not offer evidence on the observable features (ie, text, speech, or facial expression) of depression. Since we aimed to provide a complete overview of all the research activity related to our research questions, we did not treat the methodological quality of reviews and primary studies as a reason for exclusion [30,37].

### Study Selection

**Figure 1:**
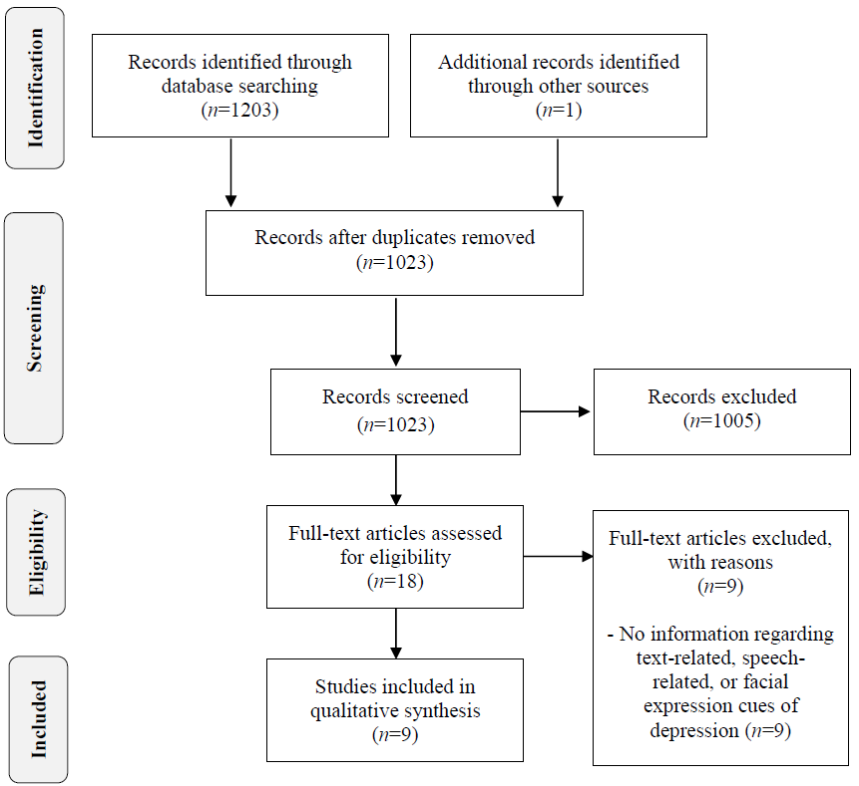
Flowchart of search and study selection.

All citations identified in the electronic databases were exported to Excel spreadsheets (Microsoft Inc). Database searches yielded a total of 1203 papers, and 1 additional paper was identified through other sources (Figure 1). After duplicates were removed, 1023 titles and abstracts were screened in 2 stages. In the first stage, the authors (IM, NP, and US) individually excluded highly irrelevant papers. In the second stage, 2 reviewers (NP and US) independently reviewed the titles and abstracts of the remaining citations and settled disagreements through discussion. After this step, 18 papers underwent a thorough full-text review independently by 2 authors. Disagreements in this phase were settled through discussion and adjudication by a third reviewer (IM). In the end, 9 papers fulfilled the predetermined criteria and were included in the scoping review.

### Charting the Data

We developed a spreadsheet form based on research questions to determine variables to be extracted from the reviewed papers. Two authors (NP and US) then extracted the following data from each paper: (1) authors, (2) year of publication, (3) type of paper, (4) number of primary studies included, (5) inclusion criteria, and (6) important results or information relevant to our research questions. During this process, results were categorized into the following categories: (1) text cues, (2) speech cues, and (3) facial expression cues. Data extraction and categorization of results were refined and updated in an iterative process as the papers were reviewed. The resulting chart was used for analysis.

### Collating, Summarizing, and Reporting Results

We did not follow a well-structured meta-analytic approach in comparing and summarizing the results given the aim and nature of a scoping review. Since the aim of a scoping review includes mapping the existing findings on a topic and providing their descriptive overview [30,37], the results were analyzed by 2 authors (NP and US) using thematic analysis [38] within 3 predefined categories of cues of depression (text, speech, and facial expression cues). To maintain rigor in collating and summarizing results, this process was reviewed by 2 authors (IM and SL).

### Consulting With Stakeholders

We developed a review protocol that ensured psychological (BM, NP, and US), medical/oncological (SL), technological (IM), and methodological (NP, US) expertise were included. Health-care professionals involved in PERSIST were consulted to develop relevant inclusion and exclusion criteria (eg, specific conditions that could impact the expression of depression).

## Results

### Characteristics of Reviewed Studies

The final selection of papers in this review (Table 1) included 2 meta-analyses (22%), 2 systematic reviews (22%), 2 literature reviews (22%), 1 scoping review (11%), and 2 empirical studies (22%) that were included because their state-of-the-art met the criteria of this scoping review. Papers were published from 1997 to 2021, with most (7/9, 78%) published after 2013. Papers included from 8 to 60 primary studies on the topics in line with this review, and most focused on more than 1 category of depression cues.

**Table 1:** An overview of the characteristics of the papers reviewed.

Reference	Type of paper	Primary studies, n^a^	Depression cue categories addressed
Bylsma et al [39]	Meta-analysis	19	Facial expression
Cummins et al [28]	Literature review	60	Facial expression, speech, other^b^
Dwyer et al [40]	Scoping review	22	Language use
Edwards and Holtzman [41]	Meta-analysis	21	Language use, speech
Kim et al [27]	Systematic review	15	Language use
Pampouchidou et al [29]	Systematic review	43	Facial expression, other
Scherer et al [42]	Review within empirical study^c^	8	Facial expression, speech, other
Shan et al [43]	Review within empirical study^c^	13	Facial expression, speech, other
Sobin and Sackeim [44]	Literature review	10	Facial expression, speech, other

^a^The number of primary studies that provided information on depression cues in 1 of 3 categories of interest.

^b^Other represents depression cues that are outside of the scope of this review.

^c^These papers include a comprehensive review and an empirical study.

### Features Related to Language Use

All 3 studies that focused on features related to language use [27,40,41] report that people with depression tend to focus on themselves, which is manifested in increased use of first-person singular pronouns, such as “I,” “my,” and "me." While the relationship between using such words and depression is relatively weak, findings of the relationship are ubiquitous and well replicated; based on their meta-analysis [41], Edwards and Holtzman suggest that it is equally present in different subsamples (eg, age, gender, clinical or nonclinical samples) and contexts (eg, written or spoken text, private or public language).

Depression can also be inferred from other linguistic features, particularly those that—either directly or indirectly—express depressive symptoms. Analyses of text-based communication have revealed that depressed individuals tend to use more negative-valence words (eg, words related to pain, expressions of sadness and anger, aggressive emotions, and rumination) and fewer positive emotion words than their nondepressed counterparts. For example, some of the specific language markers of depression include words such as ”hurt,” “tears,” ”alone,” “hate,” “sleep,” and “worry” [27,40]. Depression can also be inferred from explicit mentions of the treatment of depression (words such as “side effects” and “therapy”) [40].

Reviews [27,40] also suggest the existence of other linguistic features of depression. Patients with mental health issues, including those with depression, use more absolutist words (eg, “always,” “never”) [40] and tend to be focused on the past (eg, “learned,” “remember”) [27]. Furthermore, for example, depressed users on Twitter were found to generally have fewer words per Tweet, while other studies show that people with depression in general also exhibit lower complexity of language (ie, use fewer complex sentences) [27]. Reviews have highlighted that depressed individuals tend to avoid complex sentences containing adverbial clauses, which is a type of dependent clause that functions as an adverb (eg, “as soon as,” “even though”) [40].

### Features Related to Speech

To some extent, features related to speech overlap with features related to language use and written communication. As mentioned above, increased usage of first-person singular pronouns has been identified in spoken language [41]. Moreover, similarly to written text, depressed individuals tend to engage less in verbal communication and speak in shorter phrases [42,43]. However, spoken language is generally an even richer source of information than written text— the information that it contains includes not only the message but also the manner in which the words are spoken.

Prosody, which describes the properties of intonation, stress, and rhythm (among others), is an additional source of information that has been well studied. Reviews and studies [28,42-44] indicate that the most promising prosodic feature of depression is related to speech rate. Depressed individuals speak at a slower rate than controls (particularly at the phoneme—the smallest unit of sound in speech—level) and exhibit longer pauses when answering questions, during the conversation, and at speech initiation [28,42-44]. The speech pause time is not only a good discriminator between depressed individuals and controls but also between depressed individuals and individuals with other mental health diagnoses, including bipolar disorder [44]. Although some authors report that there are conflicting results on the effect of depression on loudness and variation in loudness [28], others emphasize that especially reduced variation in loudness seems to be a common feature of depression [42,43]. Another variable that is often studied in the context of depression is fundamental frequency (also known as pitch or F0). All papers included in our scoping review concluded that depressed individuals exhibit lower pitch variability (also known as change of pitch) compared to controls, although some authors acknowledge that there was heterogeneity in primary studies [28,42-44]. The combination of lower variation in loudness as well as lower variation in pitch can result in monotonous speech among depressed individuals [42,43]. Similarly, depression has also been linked to a lack of linguistic stress (ie, relative emphasis given to a certain syllable or word), reduced intonation [28], repetitious pitch inflections and stress patterns [43], as well as poorer articulation (specifically, in terms of diphthong production, which refers to a sound made by combining 2 vowels) [44].

Fewer papers have studied other speech-related sources of information. Studies on source features (ie, features related to the source of voice production) have primarily focused on voice quality, with results showing that depression is linked to decreased voice quality (eg, aspiration, jitter, shimmer, and breathy phonation) and that the voices of depressed individuals are generally harsher [28,42]. Several studies have focused on comparisons of the glottal spectrum and flow parameters between depressed patients and controls. For example, depressed individuals exhibit higher energy in the upper frequency bands of the glottal spectrum [28]. Depression has also been linked to increased tension in the vocal tract and the vocal folds, with differences in parameters capturing glottal flow between moderate to severe depression and speakers without depression. Differences can specifically be found in their Normalized Amplitude Quotient, which is an amplitude-based measure of the glottal flow and glottal flow derivative, and their Quasi-Open-Quotient, which refers to an amplitude-based measure of the glottal flow pulse that offers important insight into the open period of the vocal folds [28,42].

Some studies also suggest that depressed individuals differ from their nondepressed counterparts in terms of the formant (information on acoustic resonances of the vocal tract) and spectral features (information on the speech spectrum). First, depressed speech is associated with decreased formant frequencies, particularly in the second formant location (phoneme/a1), although studies also show that the first 3 formant frequencies and bandwidths, grouped together, are significantly different between depressed patients and controls [28]. Second, while spectral analysis seems to have limited usefulness in classification systems, some studies have reported a relative shift in energy from lower to higher frequency bands, while others report a reduction in subband energy variability among depressed individuals [28].

### Features Related to Facial Expression

Bylsma and colleagues [39] suggest that observing the behavioral indicators, there is reduced positive emotional reactivity in depressed patients and people with major depressive disorder compared to controls but found no differences in negative emotional response in comparison to controls. Their review also found “more pronounced blunting of positive emotional reactivity compared to negative emotional reactivity" [39]. Pampouchidou and colleagues [29] suggest that depression is associated with the variability and intensity of facial expressions (eg, reduced or decreased emotional facial expressivity [28,29,42]) with fewer animated facial expressions [43] and generally decreased facial mobility [28]. Links between depression and more frowns [29,42] and the occurrence of sad, negative, and neutral expressions [29] were also suggested.

Differences between people with depression and nondepressed individuals have been observed in eyebrow activity [29], through generally reduced eyebrow movements [28] and lower frequency and duration movements [44]. Differences can be observed in the region of the Veraguth fold (skin fold on the upper eyelid and between the eyebrows) and in the extended activity on the corrugator muscle (in the medial extremity of the eyebrow) [29].

Depression is associated with reduced saccadic eye movements [28,29], reduced horizontal pursuit [28], and increased visual fixation [28,29]. People with depression generally tend to engage less in mutual gazes [42] and limit eye contact [29]. Eye contact tends to be shorter in duration compared to normal controls [29,44] and people with schizophrenia [44] and occurs less frequently than in controls [44]. Depression tends to be associated with avoiding eye contact [43], low frequency and duration of glances [29], more gaze aversion, more downward gaze, and more nonspecific gaze [42]. Additionally, association with depression was observed in pupil dilation responses and bias, pupillary response, iris movement, and eyelid activity (eg, openings, blinking) [29].

Differences between people with depression and nondepressed individuals tend to be observed in mouth animation [29]. Specifically, they tend to present fewer mouth movements [42], more frequent lip presses, down-angled mouth corners, and reduced activity on the zygomaticus muscle (which moves the mouth angle in producing a smile) [29]. Depression is generally associated with less smiling [28] (ie, smiling less often [29,42,44] and shorter duration smiles [29,44]) and can be observed in smile intensity [29]. People with depression tend to exhibit more smile controls [42] and listening smiles (smiles presented when not speaking) [29].

Associations between depression and head pose (ie, orientation and movement) [29] were also suggested. People with depression tended to turn the head away [42] and were more likely to hold their head in a downward position [43] than nondepressed individuals. People with depression, in comparison to those with schizophrenia, tended to exhibit more large head movements and a higher occurrence and duration of small head movements [44].

## Discussion

### General

Even though cancer survivors often find themselves experiencing disorders from the depressive spectrum, these conditions frequently remain underrecognized and overlooked [4,10]. Screening for psychological distress in this population is recognized as essential, but several barriers for its successful implementation persist [13,14]. For instance, the most used method of self-reporting of symptoms can result in significant bias in reporting experiences, which is even more pronounced in people with depression [18-23]. Therefore, a better way to detect psychological disorders in cancer survivors is needed. Big data and artificial intelligence, together with a growing body of knowledge on the connection between observable cues and psychological states of a person, offer new opportunities to better detect psychological disorders in cancer survivors. As Low and colleagues [25] suggest, spontaneously generated cues of psychological states and their recognition by artificial intelligence could result in improved screening for psychological disorders.

In this scoping review, we reviewed 9 meta-analyses, systematic and literature reviews, and similar papers to conduct a meta-review of observable cues of depression. The findings of our meta-review revealed several observable characteristics of depression in each of the 3 categories. In language use, people with depression tend to show increased use of first-person singular pronouns, use more negatively valenced words, and less positive emotion words than nondepressed individuals. Additionally, the use of absolutist words, the use of words focusing on the past, and low language complexity are often present. In speech, people with depression engage in less verbal communication and speak in shorter phrases; lower speech rate, longer speech pauses, and potentially lower variation in loudness are exhibited. Some authors [42,43] also suggest lower pitch variability and change rate in people with depression, resulting in generally monotonous speech. Reduced intonation, repetitious pitch inflections and stress patterns, poorer articulation, decreased voice quality, generally harsher voice, and higher vocal tension also tend to be present. In the category of facial expressions, people with depression in comparison to nondepressed individuals show reduced positive emotional reactivity and emotional facial expressivity. Generally, they produce fewer animated facial expressions and facial mobility and exhibit more frowns and more sad, negative, and neutral expressions. There is less movement in the eyebrow region, and eyes exhibit reduced saccadic eye movements, reduced horizontal pursuit, and increased visual fixation. People with depression tend to engage in less mutual gaze, avoid or limit their eye contact, show more gaze aversion, and show more downward and nonspecific gaze. Fewer mouth movements, more lip presses, and down-angled mouth corners also proved to be evident in people with depression. Additionally, decreased smiling can be observed, together with more smile controls and listening smiles. They also turn their head away more often and hold their head in a downward position.

The diagnosis of cancer and subsequent treatments can have a large impact on patients’ psychological well-being. Along with the physical remnants of treatment, cancer survivors often continue to grapple with anxiety and depression. Almost one-third of cancer patients suffer from a comorbid mental health condition [45]. Although professional support is available after diagnosis and during treatment, the symptoms are still often overlooked in patients and long-term survivors [46], partially due to significant overlap between symptoms of emotional distress and late effects of cancer or side effects of treatment, but mainly due to depression’s complexity and subjective nature. Namely, as symptoms of depression may be normal for some, the that are symptoms present may impose significant psychological strain on other patients [47]. The difficulty in diagnosing depression in cancer patients has led to the development of several diagnostic approaches [48]. However, since screening is mostly based on self-reports during 6-month or 1-year follow-up, symptoms can remain unnoticed for a long period of time or can even be completely overlooked. The technological advances and reliability of machine learning–supported feature extraction classification methods may allow new, less intrusive, and more reliable ways to detect symptoms of depression. As highlighted in this scoping review, completely unrelated spoken or even written interactions, as well as visual cues, may reveal early warning signs that should trigger further clinical assessment.

While the technological advances may be of great benefit in recognizing these cues and signs, it is particularly important to note that many existing studies tend to exploit only 1 modality, such as text analysis [49], though it may be combined with other aspects of users’ writing (eg, the time gap between 2 consecutive writings; [50]), for artificial intelligence–based recognition of depression and other mental health issues. These approaches can be informative and helpful in the early detection of depression but tend to perform suboptimally due to their relatively narrow focus [51]. Our review shows that depression is likely expressed through all 3 forms of communication simultaneously; therefore, the prevailing unimodal approaches to artificial intelligence recognition of depression (eg, [43]) may be inadequate. Trimodal approaches are especially important in improving the accuracy of predictive models and reducing the chance of false classification of individuals as those presenting or not presenting signs of depression. For example, when using unimodal speech analysis models, an error could occur in the instance of nonnative speakers who often use first-person singular pronouns, which could be recognized as a meaningful feature of depression, whereas the result might be different when the other 2 modes are also examined. In PERSIST [26], we intend to exploit findings of this review to deliver an explainable artificial intelligence capable of sensing, detecting, and interpreting the affective states spontaneously expressed through language, speech, and mimicry during interaction (ie, diary recordings).

### Study Limitations

While this scoping meta-review offers a valuable synthesis of research on the observable cues of depression and translates this knowledge into the context of screening cancer survivors’ depression with artificial intelligence, it does not offer an all-encompassing picture of the current state of research on the behavioral features of depression. First, the papers included in our scoping review highlight that depression is also associated with cues, such as body movement (including gestures, fidgeting) and posture, that are outside of the scope of this meta-review. As these features could explain the variance in depression expressions above and beyond the features described in our review (and hence improve the accuracy of depression detection algorithms), we argue that attempts to synthesize research on these additional cues would be highly beneficial. Second, the findings of our meta-review represent the features of depression that are characteristic of the average person with depression. Future research should take into consideration potential moderators that determine whether features are present and to what extent. In other words, future research should explore which cues are relevant in which cases, to allow the development of algorithms that are robust to individuals’ characteristics. Third, because we focused only on English-language papers, based on studies that were largely conducted on Anglophone participants in industrialized countries, our conclusions may be culturally biased. Further research is thus needed to understand whether our findings can be generalized. Lastly, since we included reviews instead of original empirical papers, it is possible that we overlooked more recent papers related to this topic that have not yet been included in any reviews.

### Clinical Implications

This review provides valuable theoretical background and ideas for technological implementation that could facilitate the development of improved artificial intelligence solutions to detecting cancer survivors’ depression. We argue that such solutions may benefit clinicians as well as cancer survivors. For clinicians, these solutions may be more cost-effective and efficient for recognizing distress in patients after cancer than existing methods. As such, artificial intelligence could replace some elements of current screening procedures and supplement others. Additionally, such solutions may be used for ecological momentary assessments (and over longer periods of time), which is not possible with traditional clinical assessment and self-report instruments. For cancer survivors, on the other hand, artificial intelligence may be an accessible and nonobtrusive way of monitoring their mental health that does not require any conscious effort.

### Conclusions

Evidence from 9 reviews (based on more than 200 primary empirical studies) show that there is a robust association between depression and a wide array of specific observable cues. Such associations are an excellent theoretical underpinning for the development of artificial intelligence algorithms; therefore, it is time to move from the question of whether artificial intelligence can support the process of detecting cancer survivors’ depression to the question of how this can be done.

## Abbreviations

PERSIST: Patient-Centered Survivorship Care Plan After Cancer Treatments Based on Big Data and Artificial Intelligence Technologies
PRISMA-ScR: Preferred Reporting Items for Systematic Reviews and Meta-analyses Extension for Scoping Reviews
